# Effect of Pre- and Perinatal Factors and Infant Nutrition on the Intestinal Microbiota

**DOI:** 10.3390/nu15183977

**Published:** 2023-09-14

**Authors:** Beata Łoniewska, Igor Łoniewski

**Affiliations:** 1 Department of Neonatology and Intensive Neonatal Care, Pomeranian Medical University in Szczecin, 70-111 Szczecin, Poland; beata.loniewska@pum.edu.pl; 2 Department of Biochemical Science, Pomeranian Medical University in Szczecin, 71-460 Szczecin, Poland; 3 Department of Human Nutrition and Metabolomics, Pomeranian Medical University in Szczecin, 71-460 Szczecin, Poland; 4 Sanprobi sp. z o.o. sp. k., 70-535 Szczecin, Poland

The intestinal microbiota is an essential determinant of human health. It is responsible for food digestion and plays a significant role in human development and metabolic, immunological, and psychological processes. Research on the gut microbiota of infants and young children still needs to be completed. Children are already in contact with microorganisms in foetal life, and these interactions increase rapidly after birth and during breastfeeding. The increasing number of caesarean section births, the application of antibiotics and other drugs, and formula feeding undoubtedly affect the baby’s microbiome and may influence future health. Therefore, studying factors that may affect the microbiome, the immune system, or the intestinal barrier in early life is essential. It is also important to implement early prevention and modification of the microbiome to prevent adverse consequences from its alterations. In this Special Issue, five articles were published that analysed the influence of various factors during pregnancy, childbirth, and the first years of life on the composition and metabolic functions of the gut microbiota. The gut microbiome changes composition and function during the first 2–3 years of life [1,2]. The most crucial factor involved in the maturation of the microbiome is nutrition. Breast milk feeding has a more favourable effect on the maturation of the microbiome than formula feeding [3,4,5,6]. Infant gut bacterial colonisation is also affected by other factors, including caesarean section [7], antibiotics treatment [8], and maternal body mass index (BMI) [9]. Like with formula feeding, these factors are associated with early gut maturation, which can have an unfavourable effect on the development of the immune system [10,11] and can increase the risk of obesity [9,12]. An essential function of the microbiota is synthesising metabolites, including short-chain fatty acids (SCFAs) [13]. The most important are acetate (C2), propionate (C3), and butyrate (C4) [14]. SCFAs play an important role as a source of energy for various cells [15] and take part in gluconeogenesis and lipogenesis. The aim of the work of Łoniewska et al. [16] was to analyse the influence of perinatal factors, which can affect the gut microbiota, on the concentration of SCFAs in the stool during the first two years of life. The results show dynamic changes in the stool concentrations of SCFAs in children during the first year of life, followed by the stabilisation of their stool concentrations. In addition, the maternal and foetal factors studied, such as antibiotic intake during pregnancy, feeding method, delivery method, pre-pregnancy weight, changes in gestational weight, and children’s weight and sex, influenced the excretion of SCFAs in the stool. However, the study could not determine the long-term health consequences of these observations.

Nevertheless, it can be speculated that breastfeeding and proper weight control in mothers and children benefit health by influencing the composition of SCFAs in the stool and, thus, the development of the gut microbiota. In contrast, antibiotic therapy during pregnancy and caesarean section may harm the functioning of the gut microbiota. However, these speculations should be treated cautiously, and long-term epidemiological studies are warranted to further determine the mechanisms of these changes.

One of the predominant SCFAs—propionate—has antihypertensive properties. In a study on rats, Tain et al. [17] showed that perinatal propionate supplementation in offspring could prevent hypertension induced by the mother’s chronic kidney disease (CKD). The protective effect of propionate was associated with an increased abundance of Clostridium spp. bacteria (which produce propionate) and propionate concentrations in the animals’ plasma, an increased expression of renal G protein-coupled receptor 41 (GPR41, SCFA receptor), increased α-diversity, and changes in the intestinal microbiota composition. The results indicate that CKD-induced hypertension in the offspring can be prevented by SCFA metabolites in early life, which may be helpful for further research on this subject. 

Another experimental study analysed the impact of the mother’s metabolism during early pregnancy, which can affect the child’s emotional and behavioural processes, including vulnerability to bullying. This phenomenon results in social anxiety and increases the risk of personality disorders in bullies and their victims. The substrate of this phenomenon is not precisely known, and the influence of genetic, gestational, and environmental factors is suggested [18]. It has been observed that children born to stressed mothers are more prone to bullying [19], which may be associated with increased susceptibility to stress in people with gut microbiota disorders [20]. One of the factors influencing gut microbiota disorders in children and brain development is placental tryptophan fluctuations, which can lead to adverse physiological and socio-behavioural consequences. Huang et al. [21] investigated the hypothesis that the administration of tryptophan during pregnancy could affect children’s mental and physical development by modifying the microbiota–gut–brain axis (MGB), which underlies their mental state. Chicken embryos, whose microbiota are robust and independent from maternal influence, were used for this experiment. The results showed that exposure to tryptophan during the embryonic period reduced the male offspring’s body weight and aggressiveness before and during puberty. In addition, beneficial anatomical changes were found in the gut under the influence of tryptophan. The mechanism for these changes may involve tryptophan-induced reprogramming of the MGB axis, which causes changes in the composition of the intestinal microbiota that may result in increased synthesis of scatole and butyrate, as well as elevated concentrations of catecholamines in the thalamus and hypothalamus. These changes may underlie bullying and warrant further investigation into the possibility of dietary modification and tryptophan supplementation during pregnancy.

Another two studies focused on preterm infants, whose microbiota are less diverse and often enriched with potential pathogens (e.g., members of the Enterobacteriaceae family). In addition, preterm infants are often treated with antibiotics, destabilising the microbiota and increasing the risk of fungal infections. In an experimental study, dietary supplementation of preterm infants with medium-chain triglyceride (MCT) oil was shown to reduce the fungal load of Candida spp. in the gastrointestinal tract [22]. The study of Romo et al. [23] aimed to determine whether MCT supplementation affected the bacterial component of the microbiome. They found that MCT supplementation did not significantly alter the diversity or composition of the gastrointestinal microbiota. Notably, there were no significant changes in the Enterobacteriaceae family, suggesting that MCT supplementation did not enrich the abundance of potential pathogens. MCTs represent a promising therapeutic intervention to reduce fungal colonisation without significantly affecting the bacterial composition of the host gastrointestinal tract. Marousez et al. [24] investigated the impact of consuming early postnatal prebiotic fibre (PF) on growth, intestinal morphology, and the microbiota during weaning in postnatal-growth-restricted mice (PNGR). They found that the PNGR mice had a decreased body weight and ileal crypt depth during weaning compared to the control group and microbiota alterations. The propionate concentrations were also increased with PNGR. PF supplementation did not impact intestinal morphology in the PNGR pups but affected gut microbiota. Moreover, PF supplementation increased the Akkermansia genus in the PF-supplemented control pups compared to the water-supplemented ones. The authors concluded that PF supplementation might improve gut microbiota establishment during the early postnatal period. 

Summarising the observed results, the microbiota has an essential metabolic function during early human development. Particular caution is warranted when using drugs, in medical procedures, and in health care during pregnancy, as all of these factors can affect it. On the other hand, early intervention to modify the microbiota in this phase of life can have long-term beneficial health effects. For this reason, despite methodological difficulties, prospective studies should be conducted to assess the distant consequences of altering the microbiota early in life.
